# Infection control in dental health care during and after the SARS‐CoV‐2 outbreak

**DOI:** 10.1111/odi.13408

**Published:** 2020-05-25

**Authors:** Catherine M. C. Volgenant, Ilona F. Persoon, Rolf A. G. de Ruijter, J. J. (Hans) de Soet

**Affiliations:** ^1^ Department of Preventive Dentistry Academic Centre of Dentistry Amsterdam (ACTA) University of Amsterdam and Vrije Universiteit Amsterdam Amsterdam The Netherlands; ^2^ Expert Group Behavioral and Contemplative Dentistry of the University Medical Center Groningen/Center for Dentistry and Oral Hygiene Rijksuniversiteit Groningen Groningen The Netherlands

**Keywords:** dentistry, infection control, infectious disease transmission, Public Health Dentistry, SARS‐CoV‐2

## Abstract

COVID‐19 is an emerging infectious disease caused by the widespread transmission of the coronavirus SARS‐CoV‐2. Some of those infected become seriously ill. Others do not show any symptoms, but can still contribute to transmission of the virus. SARS‐CoV‐2 is excreted in the oral cavity and can be spread via aerosols. Aerosol generating procedures in dental health care can increase the risk of transmission of the virus. Due to the risk of infection of both dental healthcare workers and patients, additional infection control measures for all patients are strongly recommended when providing dental health care. Consideration should be given to which infection control measures are necessary when providing care in both the current situation and in the future.

## 1 INTRODUCTION

In recent months, the world has been confronted with an outbreak of the SARS‐CoV‐2 virus (Rabi, Al Zoubi, Kasasbeh, Salameh, & Al‐Nasser, 2020), resulting in the *coronavirus disease 2019*; COVID‐19 (Khan et al., 2020). This coronavirus has a single‐stranded positive RNA chain, surrounded by a capsid (Adhikari et al., 2020; Ashour, Elkhatib, Rahman, & Elshabrawy, 2020). In most cases, the virus causes mild‐to‐severe respiratory complaints (Chen et al., 2020; Huang, Wang, et al., 2020). Since the population did not have contact with this virus before, no herd immunity against the virus has been acquired among the population. In addition, the virus is highly contagious, with one person infected with SARS‐CoV‐2 infecting on average 2–3 people (reproduction number R_0_) (Liu, Gayle, Wilder‐Smith, & Rocklöv, 2020; Sun et al., 2020). This results in a potentially large group of infected individuals. By isolating these infected individuals and applying sufficient infection control measures, the R_0_ will decrease. When the R_0_ is less than 1, an outbreak will extinguish spontaneously (Chen, 2020; Heesterbeek & Dietz, 1996). However, when infected individuals show little or no symptoms, they are likely to be missed and that they will be exempted from isolation. This can contribute to the further dissemination of the virus (Munster, Koopmans, van Doremalen, van Riel, & de Wit, 2020).

The SARS‐CoV‐2 virus uses the membrane‐bound angiotensin‐converting enzyme 2 (ACE2) receptor of the host to penetrate the cells (Xu et al., 2020). The membrane‐bound ACE2 receptor is mainly found on cells of the mucosal tissues, such as the dorsum of the tongue and salivary glands (Liu et al., 2011; Xu et al., 2020). Hence, saliva has been suggested for non‐invasive diagnostics of this virus (Khurshid, Asiri, & Al Wadaani, 2020; Sabino‐Silva, Jardim, & Siqueira, 2020). The mucosal membrane of the oral cavity, respiratory tract and eyes is an important *portal of entry* for this virus (Adhikari et al., 2020; Zhang, Zhang, & Wang, 2020). This *portal of entry* also serves as a reservoir from which transmission may occur to other individuals, for example coughing or sneezing (Adhikari et al., 2020). Now the outbreak has become a pandemic and no drugs or vaccines are yet available, infection control measures are the only option for the time being to slow down the number of new infections (Lai et al., 2020). These precautions include social distancing and isolation which prompted to provide only limited emergency (dental) health care during the beginning of the outbreak in most countries (Farooq & Ali, 2020; Guo, Zhou, Liu, & Tan, 2020; Izzetti, Nisi, Gabriele, & Graziani, 2020; Meng, Hua, & Bian, 2020b; Peng et al., 2020; Spagnuolo, De Vito, Rengo, & Tatullo, 2020). In time, dental health care should be slowly upscaled beyond emergency care in order to prevent and treat oral diseases. This paper summarises the infection control measures for the dental healthcare setting in relation to SARS‐CoV‐2, based on the currently available scientific evidence.

## 2 INFECTION CONTROL IN DENTAL HEALTH CARE

Infection control in dental health care is about calculating risks; it is not possible to completely exclude all risks (Volgenant & de Soet, 2018). At first, infection control guidelines in dental health care aimed to prevent transmission of blood‐borne diseases (Kohn et al., 2003). Unique to the dental healthcare setting is the profound production of aerosols during most treatment procedures. Aerosols are liquid or solid particles in the air, which can be responsible for the transfer of micro‐organisms (Zemouri, de Soet, Crielaard, & Laheij, 2017). Considering the risk of aerosol transmission in dental health care, most patients are considered healthy and thus less strict aerosol precautions have to be taken compared to general healthcare settings (Siegel, Rhinehart, Jackson, Chiarello, & Committee, 2007). As a result, dental health care has always been provided in such a way that it has a relatively limited effect on feasibility and costs. Current guidelines in dental health care worldwide strive for *optimal* and feasible rather than *maximal* precautions.

## 3 TRANSMISSION OF CORONAVIRUS SARS‐CoV‐2

It is suggested that four categories of transmission of SARS‐CoV‐2 occur: symptomatic transmission (direct transmission from a COVID‐19 patient), presymptomatic transmission (direct transmission from a SARS‐CoV‐2‐positive person without symptoms yet), asymptomatic transmission (direct transmission from a SARS‐CoV‐2‐positive person who never develops symptoms) and environmental transmission (indirect transmission which is not traceable to an index patient) (Ferretti et al., 2020). Consequently, during the COVID‐19 pandemic, patients in the dental healthcare setting cannot be considered healthy even when they are not experiencing symptoms. Transmission of the SARS‐CoV‐2 virus has been reported from 12.6% of the patients before they showed symptoms (Du et al., 2020). Presymptomatic transmission from an index patient has been estimated to account for up to 44% of total transmissions and even occurred up to 2 days before symptoms were experienced (He et al., 2020). The time between infection and symptom onset (incubation period) of COVID‐19 is on average 5.1 days (CI 95% 4.5–5.8), up to a period of 11.5 days (CI 95% 8.2–15.6) (Lauer et al., 2020). Numerous publications reported on transmission during the incubation period of COVID‐19 (Huang, Xia, Chen, Shan, & Wu, 2020; Tong et al., 2020; Yu, Zhu, Zhang, & Han, 2020).

Several studies described individual cases of asymptomatic individuals (Arons et al., 2020; Chan et al., 2020; Hoehl et al., 2020; Kimball, 2020; Luo et al., 2020; Pan et al., 2020; Zou et al., 2020), as well as transmission from an asymptomatic index (Bai et al., 2020; Rothe et al., 2020). The viral load did not differ between symptomatic and asymptomatic carriers (Zou et al., 2020). In addition, mild symptoms or loss of smell or taste are also recognised as possible symptoms of infection with SARS‐CoV‐2 (Bai et al., 2020; Hu et al., 2020; Lai et al., 2020), making it difficult to identify a true asymptomatic individual. There could be a potential large reservoir of individuals in the population (Verity et al., 2020) that can contribute to rather hidden ongoing transmission (Munster et al., 2020).

The case fatality rate of this virus, reported in China, is 3%–11% (Rajgor, Lee, Archuleta, Bagdasarian, & Quek, 2020). In addition, often only mild symptoms occur (Kluytmans et al., 2020; Verity et al., 2020). Infected persons can thus continue to participate in the population, making it likely that dental healthcare workers (DHCWs) will come into contact with SARS‐CoV‐19‐positive patients (Cheng et al., 2020). However, the case fatality rate is based on the number of deaths per confirmed case. The infection fatality rate of the virus has been estimated for the whole population of China at 0.66% (Verity et al., 2020). It is likely that both the infection fatality rate and the case fatality rate will change in the coming period when more tests will be performed.

## 4 TRANSMISSION OF SARS‐CoV‐2 IN DENTAL HEALTH CARE

**FIGURE 1:**
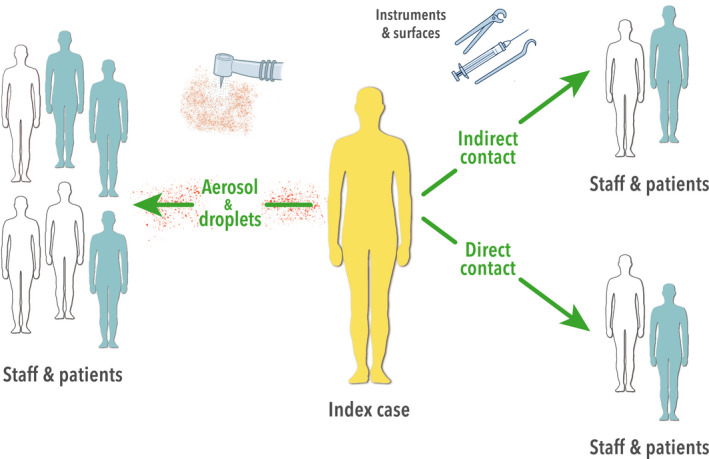
Transmission of SARS‐CoV‐2 can occur via direct contact, indirect contact and via air (droplets/aerosols). This can take place from patients to the DHCWs and vice versa, and reciprocal between patients or DHCWs. This applies to symptomatic, presymptomatic and asymptomatic individuals

The transmission of the virus mainly occurs via respiratory droplets and faecal shedding (Liu, Ning, Chen, Guo, Liu, et al., 2020; Ngoc et al., 2020; Ong et al., 2020). These respiratory droplets are excreted from the oral cavity and pharynx, for example by speech, and usually do not reach more than 1.5–2 m (Ai & Melikov, 2018; Bischoff, Swett, Leng, & Peters, 2013). When coughing and sneezing, aerosols are also generated, in which the aerosols remain in the air for some time (Jones & Brosseau, 2015). Aerosols are able to reach beyond the social distancing instructions of 1.5–2 m, for example due to aerodynamic effects (Bischoff et al., 2013; Blocken, Malizia, van Druenen, & Marchal, 2020) and transmission of the SARS‐CoV‐2 virus via aerosols has been suggested (Liu, Ning, Chen, Guo, Liu, et al., 2020). Although aerosols do not play a major role in transmission of SARS‐CoV‐2 in most daily activities, the situation is different in the dental clinic. Water in combination with compressed air used for coolant and spraying causes aerosols which become contaminated with micro‐organisms from the oral cavity (Zemouri et al., 2017). DHCWs operate at a distance of 60 cm or less from a patient's oral cavity. A recent study indicates that the largest microbiological contamination within the dental healthcare clinic takes place within 1 m from the oral cavity, via both splashes and aerosols (Zemouri et al., 2020). In medical practice, transmission of SARS‐CoV‐2 virus via aerosols is suggested in addition to transmission of the virus via droplets (Ong et al., 2020; Wax & Christian, 2020). It appears that different types of coronaviruses can already be detected in aerosols produced by exhalation (Leung et al., 2020). Moreover, a SARS‐CoV‐2‐positive patient has many virus particles in his saliva and on the dorsum of the tongue (To et al., 2020; Xu et al., 2020). This suggests that aerosols generated during dental healthcare treatment in these individuals can also contain SARS‐CoV‐2 and thereby transmit the virus to the DHCWs (Figure 1).

Even after completing treatment, aerosols are suspended in the air within the treatment room, with heavier and larger particles settling faster (Bennett et al., 2000; Nikitin, Petrova, Trifonova, & Karpova, 2014). Settling occurs on all horizontal surfaces, after which these can act as a vehicle for transmission of the SARS‐CoV‐2 virus via indirect contact (Figure 1). Viable virus was still detectable on, for example, plastic surfaces after 72 hr, up to 7 days (van Doremalen et al., 2020). Regardless of the modes of transmission, the minimal infectious dose of SARS‐CoV‐2 has not yet been established. Therefore, irrespective of the level of contamination, all surfaces contaminated with aerosol or touched by patients should be regarded as potentially contaminated.

## 5 INFECTION CONTROL MEASURES

The dental healthcare setting can be an important route for transmission of airborne or drop‐related infectious diseases, both for the dental team and the patient (Volgenant & de Soet, 2018) (Figure 1). Whenever new infectious diseases emerge, infection control protocols within the dental health care are modified accordingly (Monaghan, 2016; Smales & Samaranyake, 2003).

**FIGURE 2:**
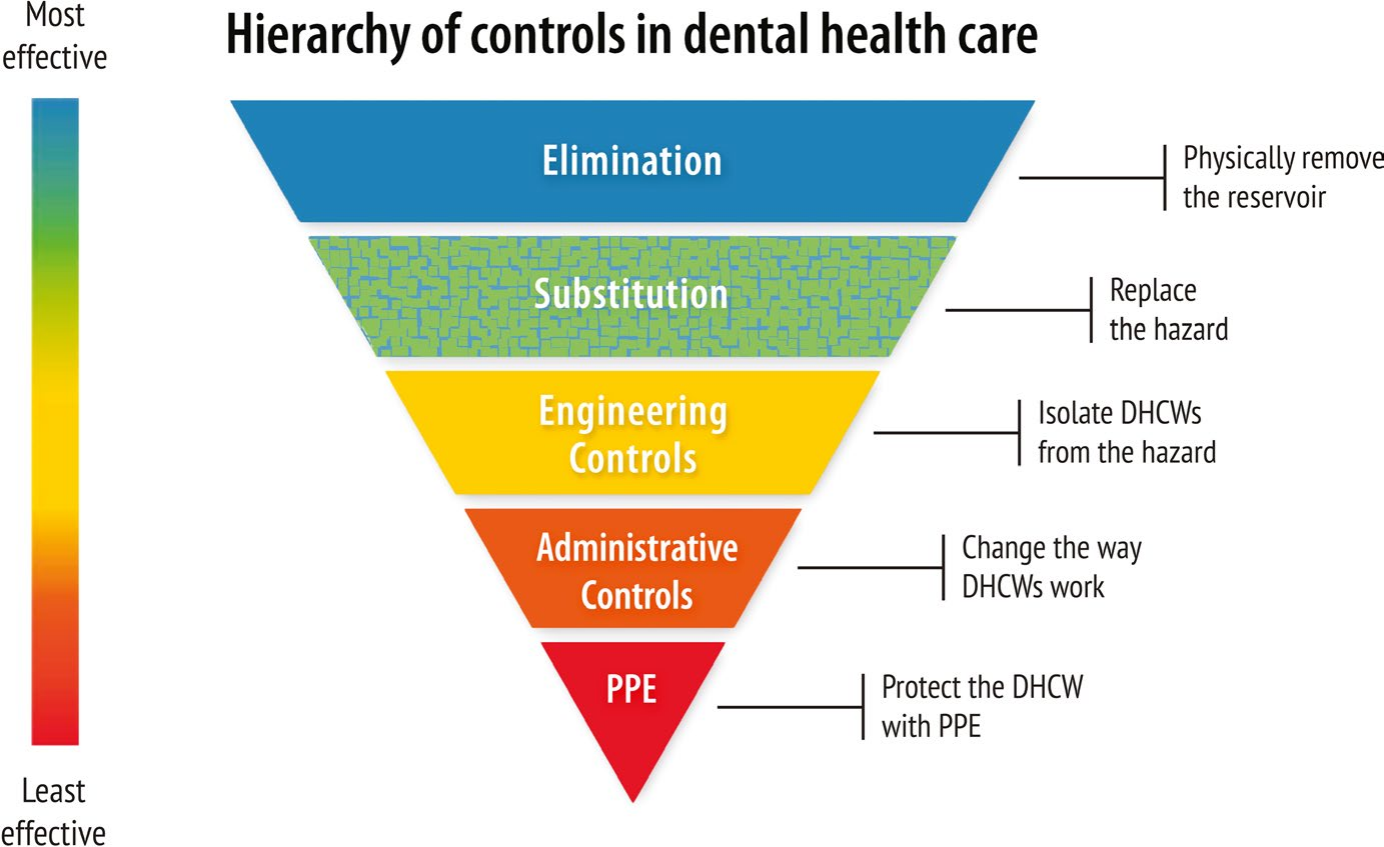
Controlling exposure to occupational risks is aimed when protecting DHCWs. The hierarchy shown here is used to determine on which level feasible and effective solutions can be implemented in the dental clinic (adapted from National Institute for Occupational Safety and Health (NIOSH, 2015) of the Centre for Disease Control and Prevention (CDC), United States)

Protection against work‐related infectious diseases can be implemented at different hierarchal levels (Figure 2). The National Institute for Occupational Safety and Health (NIOSH) in the United States conducts research and makes recommendations to prevent work‐related diseases. Measures that intervene at a level closer to the source of the virus are generally more effective and provide more protection than measures closer to the healthcare workers. The starting point of these necessary measures is that possibly all patients may be SARS‐CoV‐2 positive, although not symptomatic yet, and that SARS‐CoV‐2 is transmitted when providing dental health care, mainly via aerosols. Hence, possible adjustments to the regular protocols and the considerations involved are discussed below.

### 5.1 Elimination of the reservoir

The infectious reservoir can be eliminated by preventing contact with an infected patient. Many guidelines in infection control in dental health care are based on this principle (Kohn et al., 2003). Infected patients are assumed to be too ill to visit the dental clinic or, as a result of the anamnesis, elective care is postponed. During the SARS‐CoV‐2 outbreak, dental health care is limited to providing urgent care in most countries (Farooq & Ali, 2020; Izzetti et al., 2020; Meng, Hua, & Bian, 2020a).

- Part of the demand of dental health care can be met by telephone, email or videoconferences (Guo, Wu, & Xie, 2020; Meng et al., 2020b). A detailed patient history, possibly accompanied by photographs or video conferencing, can aid the primary management of dental emergencies. Urgent care can be managed by prescribing analgesia, antiseptics or as a last option antibiotics (Ather, Patel, Ruparel, Diogenes, & Hargreaves, 2020). These modern techniques can also be applied when providing preventive dental health care (Darwish, 2020; Mallineni et al., 2020).
- In an area where widespread transmission has not been established, triage can aid in estimating risks of transmission of SARS‐CoV‐2. Individuals with a travel history to areas with ongoing transmission or individuals with recent exposure to SARS‐CoV‐2‐positive individuals should be considered as a high risk for serving as a source of transmission. Moreover, individuals showing signs and symptoms of COVID‐19 (e.g. coughing or a fever; see above) should also be considered as high risk for transmission. DHCWs who comply with criteria as described above should be considered as high risk and therefore should not be present in the dental clinic.
- For detecting patients to who care can be provided with limited risk of transmission with SARS‐CoV‐2, triage is applicable (Alharbi, Alharbi, & Alqaidi, 2020; Ayebare, Flick, Okware, Bodo, & Lamorde, 2020; Izzetti et al., 2020; Prati, Pelliccioni, Sambri, Chersoni, & Gandolfi, 2020). Dental health care for patients showing signs and symptoms of COVID‐19 should be limited to urgent care and can only be provided in a clinic with full protective measures. DHCWs have to keep in mind that triage is currently unable to differentiate asymptomatic or presymptomatic patients from unaffected individuals. Reliable, simple and cheap rapid tests can assist in determining to who dental health care can be provided without additional measures (Khurshid et al., 2020).
- When providing care is indispensable, elimination of (secondary) infectious reservoirs is essential in preventing transmission. Both hand hygiene and hygiene of surfaces have always been important measures against the spread of viruses in society and health care; this applies to SARS‐CoV‐2 as well (Lotfinejad, Peters, & Pittet, 2020; Lynch, Mahida, Oppenheim, & Gray, 2020; Nicolaides, Avraam, Cueto‐Felgueroso, González, & Juanes, 2020; Ran et al., 2020). It is important to realise that DHCWs should prevent touching their own face, both with and without personal protective equipment (PPE) (Elder, Sawyer, Pallerla, Khaja, & Blacker, 2014). Cleaning removes the virus mechanically and disinfection inactivates the virus. Surfaces that may be contaminated with SARS‐CoV‐2 can effectively be disinfected within 1 min by applying at least 62% alcohol, 0.5% hydrogen peroxide or 1,000 ppm (0.1%) sodium hypochlorite (Kampf, Todt, Pfaender, & Steinmann, 2020). Effective disinfection with different alcohol‐based hand rub formulations and lower dilutions of alcohol has also been reported (Kratzel et al., 2020).
- The procedures for cleaning, disinfection and sterilisation of instruments can be performed as described in regularly applicable guidelines in dentistry (Kohn et al., 2003). However, mechanical cleaning is strongly recommended (automated washer disinfectors) to prevent transmission by, for example, splashing during cleaning. Cleaning and/or disinfection should also include all horizontal surfaces in the treatment room and all other items and locations in the clinic that could have been touched by the patient (Kampf, Scheithauer, Lemmen, Saliou, & Suchomel, 2020).

### 5.2 Engineering controls: isolating DHCWs from the hazard

- The air in the treatment room after an aerosol generating procedure should be regarded as contaminated. Dispersion of the virus throughout the dental clinic should be avoided, even though it is currently unknown whether the amount of virus particles in the air after an aerosol generating procedure in dental health care can exceed the infectious dose. Therefore, working under negative air pressure would be preferable (Cheong & Phua, 2006). Clean air will be drawn from less contaminated areas towards the treatment room. The active exhaust flow from the contaminated treatment room leads to removal of possible pathogens from the air.
- In most dental clinics, working under negative air pressure is not possible. Sufficient ventilation in the room (Meng et al., 2020a) will dilute the virus load (Stockwell et al., 2019). On the one hand, mechanical ventilation, possibly enhanced, can significantly increase the expulsion of air. On the other hand, natural ventilation can be improved by active ventilation and, if possible, create a draught through the room (Escombe, Ticona, Chávez‐Pérez, Espinoza, & Moore, 2019). Research data on the required duration of ventilation regarding SARS‐CoV‐2 in the dental clinic is not yet available. A case report suggested the spread of SARS‐CoV‐2 virus particles via droplet transmission prompted by air‐conditioned ventilation (Lu et al., 2020). Therefore, potentially infected air should not be transported to people in the vicinity of the clinic.
- Some procedures do not require direct patient contact, for example scheduling of appointments. Indicating at which distance interaction is recognised as safe may be considered or installing physical barriers at the front desk, for example clear partitions. The interior of the dental clinic should be assessed and if necessary rearranged to allow for maintaining a safe distance, for example rearranging the waiting area.

### 5.3 Administrative controls: changing the way DHCWs organise their work

- The routing within the dental clinic should be arranged in such way that both DHCWs and patients are able to maintain distance from each other when DHCWs are not wearing PPE. Social distancing between DHCWs should also be maintained when not caring for a patient, for example when changing clothes or during breaks.
- Many infection control measures require changes in behaviour (Kretzer & Larson, 1998). Therefore, extra attention to (the behaviour of) the team is imperative and should be aimed at creating awareness to the adjusted procedures in order to prevent contamination between DHCWs. It is important to provide them with appropriate information, education and training and to provide sufficient resources to promote the behavioural changes.
- During dental treatment, the virus load in aerosols can be reduced by applying a leakproof rubber dam (Cochran, Miller, & Sheldrake, 1989; Rørslett Hardersen, Enersen, Kristoffersen, Ørstavik, & Sunde, 2019; Samaranayake, Reid, & Evans, 1989). The work field should be disinfected after the application of rubber dam. Furthermore, apart from reducing the microbial load from aerosols, rubber dam can also contribute to reducing splashes (Dahlke et al., 2012).
- Aerosol dispersion should be minimised by adjusting dental treatment procedures, for example by using hand instruments instead of water‐cooled instruments or ultrasonic cleaning devices (Harrel, Barnes, & Rivera‐Hidalgo, 1998). In addition, adequate saliva as well as aerosol extraction using high volume evacuation is important to minimise aerosol production (Devker et al., 2012; Narayana, Mohanty, Sreenath, & Vidhyadhari, 2016). Procedures that provoke gag reflexes or coughing should be avoided if possible (Meng et al., 2020a).
- Thirty minutes after aerosol formation, virus particles and bacteria can still be detected in the air of the treatment room (Bennett et al., 2000; Nikitin et al., 2014). Transmission to unprotected DHCWs in between treatments as well as to the next patient should be prevented. Alternatively, to waiting at least 30 min between patients, sufficient ventilation may be applied (more information under Engineering controls).
- Recent publications suggested that rinsing the oral cavity with hydrogen peroxide (1% H_2_O_2_) may be useful in reducing the risk of transmission of SARS‐CoV‐2 via aerosols (Ather et al., 2020; Peng et al., 2020). However, since the viral load is high in the throat, in the nose, on the tongue and in the saliva (Liu et al., 2011; Xu et al., 2020; Zou et al., 2020), the oral cavity will soon be recontaminated after rinsing. Povidine‐iodine has been suggested to be useful for both oral and nasal disinfection against SARS‐CoV‐2 (Kirk‐Bayley, Challacombe, Sunkaraneni, & Combes, 2020; Loftus, Dexter, Parra, & Brown, 2020; Rørslett Hardersen et al., 2019). A systematic literature review reported that rinsing with all kinds of other orally applied disinfectants reduces the microbiological load in aerosols generated during dental healthcare procedures (Marui et al., 2019), but it is unclear whether this reduction is clinically relevant for prevention of SARS‐CoV‐2 transmission. In vitro studies on chlorhexidine showed that it insufficiently inactivates SARS‐CoV‐2 (G. Kampf, Scheithauer, et al., 2020).

### 5.4 Protection of the DHCW with PPE

- Since the respiratory tract is the main *portal of entry* of the virus, the respiratory tract should be shielded (Jin et al., 2020). Therefore, the recommendation is to wear respiratory protection during aerosol generating procedures in patients infected with SARS‐CoV‐2 (WHO, 2020). These respiratory protective devices (filtering half masks: FFP‐2/ N95/ KN95) filter particles significantly more effectively and have a better fit compared to regular medical face masks (type IIR, fluid resistant). PPE should protect the patient as well as fellow DHCWs against the micro‐organisms exhaled by the user. Therefore, a mask with an exhalation valve should not be worn in dental health care, as it does not protect against splashes and respiratory micro‐organisms from the user are released via the valve. It is essential that PPE complies with international standards for example European standard EN 149:2001 + A1: 2009 for respirators (British_Standards_Institution, 2011, 2019). In research on protection against fine particles, N95‐equivalent respirators showed 9% total leakage, whereas for medical face masks leakage was 22%–35% (Steinle et al., 2018). It should be noted that medical face masks are designed to protect the patient against the exhaled air from the DHCW and do not protect DHCWs against aerosols. In a systematic review, the use of respirators was compared with medical face masks and was not associated with a lower risk of laboratory‐confirmed influenza. The authors therefore suggested that these respirators should not be recommended for general public or non‐high‐risk medical staff, who are not in close contact with influenza patients or suspected patients (Long et al., 2020). Moreover, the effectiveness of respirators strongly depends on the proper intended use (Noti et al., 2012). The use of respirators significantly reduces the risks, but does not completely eliminate them. Clinical studies on the efficacy of masks in dentistry concerning virus protection have not been performed yet. The availability and prioritisation of PPE may influence which protection may be used within a dental clinic.
- The mucous membranes of the eyes are also a possible *portal of entry* (Adhikari et al., 2020). Therefore, goggles or a face shield should be used during treatment. The advantage of a face shield is its protection of mask from splashes (Lindsley, Noti, Blachere, Szalajda, & Beezhold, 2014).
- Transmission via surfaces like clothing can be prevented by careful behaviour (no touch) or by wearing a splash‐proof long‐sleeved apron over standard protective clothing. This apron should be considered contaminated after an aerosol generating treatment and should not be touched during treatment and should be discarded immediately after leaving the treatment room. All skin and other body parts left uncovered when wearing PPE should be carefully covered (wearing water resistant caps) or cleaned and/or disinfected afterwards (shoes, hair). An intact skin serves as a proper barrier against the SARS‐CoV‐2 virus, but can also serve as a vector for transmission. Hence, hygiene of DHCWs other than their hands is also required.

## 6 FUTURE PERSPECTIVES FOR DENTAL HEALTH CARE

The first phase of the outbreak required providing only urgent dental health care. Gradually, elective dental health care can be resumed, only on the condition of applying additional infection control measures combined with a risk assessment per patient. DHCWs and patients are at an increased risk. The modified procedures described in this paper aim towards a *maximal* effect instead of an *optimal* effect. These additional precautions have been effective in preventing new infections with SARS‐CoV‐2 when providing emergency dental care in China (Meng et al., 2020a, 2020b).

In Europe, several standard guidelines for infection control in dental health care have already been adapted to the SARS‐CoV‐2 pandemic. So far, these guidelines assume a reliable triage as well as a negligible number of asymptomatic or presymptomatic patients. Currently, insufficient original research is available on both the virus (e.g. on its infectivity and the minimal infection dose) and the specific risks of aerosol generating dental procedures. Given this uncertainty, the duty of care towards patients should be balanced with both the safety of DHCWs and patients, as well as the limited resources of both time and materials (especially respirators) (Kampf, Scheithauer, et al., 2020) and finally the economic consequences. In most countries, decisions regarding these issues are made by governmental institutions and are beyond the scope of this paper. However, the authors stress the importance on being open to all DHCWs to what extent the risks are unknown.

When gradually scaling down additional preventive measures, risks and feasibility should be carefully balanced. The pandemic, possibly followed by postpandemic outbreaks, will likely remain present for a prolonged period of time (Kissler, Tedijanto, Goldstein, Grad, & Lipsitch, 2020). Since it is impossible to perform social distancing in dental health care, it is required to continuously consider which precautions are needed, possibly for the coming years. Especially regarding the risk assessment of aerosol generated during dental health care, future research should focus on determining which measures are adequate for providing safe care.

## CONFLICT OF INTEREST

None to declare.

## AUTHOR CONTRIBUTIONS

**Catherine Volgenant:** Conceptualization; Writing‐original draft. **Ilona F. Persoon:** Conceptualization; Writing‐original draft. **Rolf A.G. de Ruijter:** Conceptualization; Writing‐review & editing. **J. J. de Soet:** Conceptualization; Writing‐original draft.
